# Clinical and genetic findings in a Chinese family with *VDR*-associated hereditary vitamin D-resistant rickets

**DOI:** 10.1038/boneres.2016.18

**Published:** 2016-06-21

**Authors:** Qianqian Pang, Xuan Qi, Yan Jiang, Ou Wang, Mei Li, Xiaoping Xing, Jin Dong, Weibo Xia

**Affiliations:** 1 Department of Endocrinology, Key Laboratory of Endocrinology, The Ministry of Health, Peking Union Medical College Hospital, Chinese Academy of Medical Sciences, Beijing, China; 2 Department of Endocrinology, The First Affiliated Hospital of Shanxi Medical University, Taiyuan, China

## Abstract

Hereditary vitamin D-resistant rickets (HVDRR) is a rare autosomal recessive disorder characterized by severe rickets, hypocalcemia, hypophosphatemia, secondary hyperparathyroidism, and elevated alkaline phosphatase. This disorder is caused by homogeneous or heterogeneous mutations affecting the function of the vitamin D receptor (VDR), which lead to complete or partial target organ resistance to the action of 1,25-dihydroxy vitamin D. A non-consanguineous family of Chinese Han origin with one affected individual demonstrating HVDRR was recruited, with the proband evaluated clinically, biochemically and radiographically. To identify the presence of mutations in the *VDR* gene, all the exons and exon–intron junctions of the *VDR* gene from all family members were amplified using PCR and sequenced. The proband showed rickets, progressive alopecia, hypocalcemia, hypophosphatemia, secondary hyperparathyroidism, and elevated alkaline phosphatase. She also suffered from epilepsy, which is rarely seen in patients with HVDRR. Direct sequencing analysis revealed a homozygous missense mutation c.122G>A (p.C41Y) in the *VDR* gene of the proband, which is located in the first zinc finger of the DNA-binding domain. Both parents had a normal phenotype and were found to be heterozygous for this mutation. We report a Chinese Han family with one individual affected with HVDRR. A homozygous missense mutation c.122G>A (p.C41Y) in the *VDR* gene was found to be responsible for the patient’s syndrome. In contrast to the results of treatment of HVDRR in other patients, our patient responded well to a supplement of oral calcium and a low dose of calcitriol.

## Introduction

Hereditary vitamin D-resistant rickets (HVDRR; OMIM 274400) is a rare, autosomal recessive disorder characterized by severe rickets, hypocalcemia, secondary hyperparathyroidism, hypophosphatemia, and elevated alkaline phosphatase. Approximately 80% of patients with HVDRR have early-onset alopecia, either totalis or partialis, as the degree of alopecia is associated with the severity of the vitamin D resistance. The hallmark of the disease is hypocalcemia despite elevated 1,25-dihydroxy vitamin D [1,25(OH)_2_D] level, implying resistance to 1,25(OH)_2_D.^1^ 1,25(OH)_2_D, the hormonally active form of vitamin D, which binds to the vitamin D receptor (VDR) to modulate its actions through altering expression of target genes, is essential for calcium homeostasis and bone formation. HVDRR is caused by homogeneous or heterogeneous mutations affecting the function of VDR, which lead to a complete or partial resistance to the action of 1,25(OH)_2_D (ref 2) in target organs.

The *VDR* gene is located on chromosome 12q13.11 (GenBank accession no. NG_008731.1) and encodes a predicted 427-amino-acid protein, VDR (GenBank accession no. NP_000367.1), which belongs to the steroid–thyroid–retinoid receptor gene superfamily of nuclear receptors. The structure of the VDR protein is similar to that of other nuclear receptors, which includes an N-terminal A/B regulatory domain containing the activation function-1 region, DNA-binding domain (DBD), hinge domain, ligand-binding domain (LBD), and activation function-2 region.^3^ To date, a total of 45 mutations in the *VDR* gene have been reported as the cause of HVDRR, including missense mutations, nonsense mutations, and splicing mutations. Most of the pathogenic mutations are located in the DBD and LBD. DBD mutations prevent the VDR from inducing gene transcription even though 1,25(OH)_2_D binding to the VDR is normal. In contrast, mutations in the VDR LBD have been shown to interfere with hormone binding or heterodimerization with retinoic acid X receptor, leading to complete or partial hormone insensitivity.^4^

HVDRR patients previously reported are mainly from Middle East countries and west Asia,^5^ with only one case reported in China.^6^ The cumulative data indicate that the major therapeutic approach of HVDRR is oral supraphysiological doses of calcitriol and calcium or intravenous infusion of calcium. Nevertheless, this treatment must be continued for a long period and often fails to improve patients quality of life.^7,8^ In this study, we reported a Chinese girl who presented with typical clinical and biochemical features of HVDRR, as well as epilepsy. Sequencing analysis of her genomic DNA showed the presence of a known homozygous mutation (c.122G>A, p.C41Y) in the DBD of the *VDR* gene.^9^

## Materials and methods

### Subjects

In the present study, a family of Chinese Han origin with one patient demonstrating HVDRR was recruited. The proband was a 13-year-old girl who was diagnosed with HVDRR in the Department of Endocrinology of Peking Union Medical College Hospital (PUMCH) on the basis of clinical, biochemical, and imaging findings. The parents were non-consanguineous and both showed normal phenotype. Informed consents and approval by the local ethics committee at PUMCH were obtained before the study.

### Biochemical parameters

Fasting blood samples of the proband were stored at room temperature for 30 min and centrifuged at 3 000 r·min^−1^ for 10 min to separate the serum for analysis. Age and sex appropriate reference ranges were obtained from the central laboratory of PUMCH. The levels of serum phosphate (Pi), calcium (Ca), alkaline phosphatase (ALP), and other biochemical parameters were analyzed spectrophotometrically using routine assays available at the central laboratory of PUMCH. Serum 25-hydroxyvitamin D [25(OH)D] and intact parathyroid hormone were determined by an automated Roche electrochemiluminescence system (E170; Roche Diagnostics, Basel, Switzerland), whereas serum 1,25(OH)_2_D level was determined by a 1,25(OH)_2_D ^125^I RIA kit (DiaSorin, Stillwater, Minnesota, USA) at the central laboratory of PUMCH.

### Bone mineral density

The bone mineral density (BMD) of the lumbar spine vertebrae 1–4 (L1–L4) and the left proximal femur, including the femoral neck and total hip, were measured by dual-energy X-ray absorptiometry (GE Lunar, Madison, Wisconsin, USA) at the Department of Radiology of PUMCH. The height and weight of the participants were measured with standardized equipment.

### Molecular genetic analysis

Whole-blood samples were drawn from the three family members. Genomic DNA was extracted from peripheral white blood cells using a commercial DNA extraction kit (QIAamp DNA; Qiagen, Hilden, Germany) according to the manufacturer’s instructions. Exons 2–9 of the *VDR* gene were amplified with eight pairs of primers according to a standard PCR protocol. The primers were designed using Gene Runner Primer Analysis Software (Provided by Frank Buquicchio and Michael Spruyt; http://www.generunner.net/) (Supplementary Table 1). The amplified products were sequenced using an automated sequencer (ABI3730XL) according to the manufacturer’s protocol. Sequence alignment was performed using the Basic Local Alignment Search Tool (Blast) of the National Center for Biotechnology Information database. The identified *VDR* mutation was subsequently investigated in her parents by the same method, and also analyzed in 50 unrelated Chinese Han subjects, who volunteered for an epidemiological investigation of osteoporosis throughout the country.

Finally, the mutation in the *VDR* gene was studied at the protein level. Protein modeling was conducted using the data of VDR structure in the Protein Data Bank (PDB ID: 1YNW, http://www.rcsb.org), and mutation-related residues were positioned in the three-dimensional structural model using the Swiss-PDB Viewer (Provided by Guex N and Peitsch MC, http://www.expasy.org/spdbv/).

## Results

### Patient characteristics

The proband is a Chinese girl who is the only child of her non-consanguineous parents, with no family history of rickets. She had a full-term natural delivery with a birth weight of 3.3 kg. At birth, she had normal hair, but she was found to have progressive loss of hair from the scalp and eyebrows to almost alopecia totalis from the age of 1 month. As she started to walk at 11 months of age, her motor development seemed to be normal. At 2-year old, she was diagnosed with hypophosphatemia rickets because of progressively deformed lower limbs, hypophosphatemia (0.6 mmol·L^−1^, reference range 1.29–1.94 mmol·L^−1^), and elevated alkaline phosphatase (632 IU·L^−1^, reference range 20–220 IU·L^−1^). She was given oral phosphate (75 mL per day) and alfacalcidol (0.5 μg per day). Despite receiving regular treatment, she often had hypocalcemictetany. Periodical biochemical evaluations showed hypocalcemia with serum calcium between 1.66 and 2.40 mmol·L^−1^, hypophosphatemia with serum phosphate between 0.6 and 1.02 mmol·L^−1^, and elevated alkaline phosphatase between 524 and 906 U·L^−1^. During this period of vitamin D supplementation, the dosage of alfacalcidol remained the same, but was not adjusted based on the serum calcium, phosphate, and ALP levels. She had suffered from epilepsy since the age of 10 years, which presented as a sudden impairment of consciousness and heterotropy with abrupt onset and termination. Each episode lasted for tens of seconds to a few minutes. Her electroencephalogram showed generalized 3-Hz spike wave activity during one episode, which indicated a medium abnormal discharge of neurons in the brain. She was treated with lamotrigine (200 mg per day) and the frequency of the episodes diminished.

**Figure 1:**
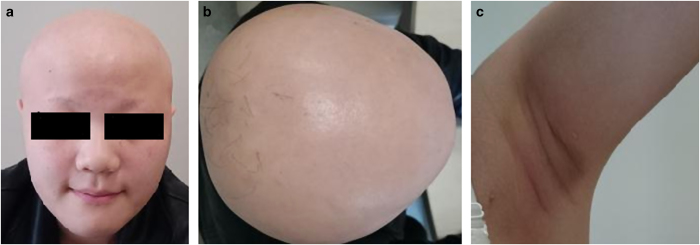
Clinical manifestations of the Chinese patient with HVDRR (**a**–**c**) showed almost alopecia totalis with few eyebrows, eyelashes, and glandebalae, respectively.

She first came to our clinic at 13 years of age because of growth retardation, with a height of 144 cm (less than −3 s.d.) and weight of 43 kg (less than −1 s.d.). Physical examination showed alopecia totalis with few eyebrows, eyelashes, and glandebalae (Figure 1). The thyroid was noticed to have primary enlargement with no tenderness or nodes. She also had signs of rickets such as rachitic rosary, costal margin eversion and genu varum. The distance was 4 cm between her knees and 6.5 cm between her ankles. The BMD values of the patient at L1–L4 and hip sites are shown in Table 1, and the *Z* score of the patient was calculated by comparison with BMD measurements from age-matched Chinese children.^10^

### Biochemical findings

Biochemical examinations of the proband were reviewed before regular treatment (Table 2). They revealed hypocalcemia (1.85 mmol·L^−1^, reference range 2.13–2.70 mmol·L^−1^), hypophosphatemia (0.80 mmol·L^−1^, reference range 1.29–1.94 mmol·L^−1^), elevated serum intact parathyroid hormone (545.6 pg·mL^−1^, reference range 12.0–65.0 pg·mL^−1^), ALP (540 U·L^−1^, reference range 58–400 U·L^−1^), 1,25(OH)_2_D (358.4 pg·mL^−1^, reference range 19.6–54.3 pg·mL^−1^) and normal levels of 25(OH)D (12.1 ng·mL^−1^, reference range 8.0–50.0 ng·mL^−1^). We noticed the elevated 1,25(OH)_2_D concentration with hypocalcemia, secondary hyperparathyroidism, and rickets, which suggested the diagnosis of HVDRR. She was given calcitriol 0.75 μg per day and calcium 1 200 mg per day. During her 2 years of therapy, her manifestations of rickets improved and there were no more episodes of hypocalcemictetany. Her serum calcium level rose to 2.40 mmol·L^−1^, and her serum phosphate to 1.28 mmol·L^−1^. Her serum intact parathyroid hormone went down to 74.1 pg·mL^−1^, ALP to 141 U·L^−1^, and her serum 1,25(OH)_2_D ranged from 358 to 200.4 pg·mL^−1^, which was measured immediately after administration of vitamin D (calcitriol 0.75 μg per day and calcium 1 200 mg per day). Radiographic examination of both knees showed only osteopenia of the distal ends of the long bones; widening of the epiphyseal cartilage and irregular cartilage ossification were not shown. However, alopecia persisted without obvious improvement.

The proband’s parents were non-consanguineous, without rickets, alopecia, or any other abnormal clinical signs.

### Genetic analysis

**Figure 2:**
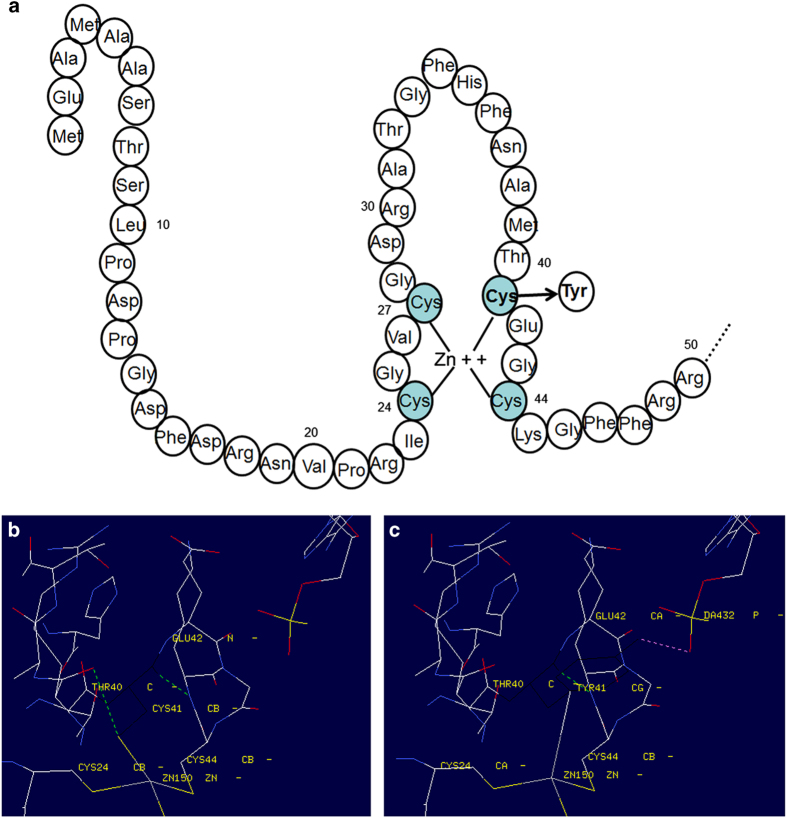
Topological model for the first zinc-finger structure of VDR DNA-binding domain and the three-dimensional structural model of VDR constructed by the Swiss-PDB Viewer. (**a**) Schematic diagram of the VDR DNA-binding domain and first zinc-finger structure. The location of the C41Y mutation is indicated in bold. (**b**, **c**) Close-up of the three-dimensional structural model of VDR using the Swiss-PDB Viewer. (**b**) Position 41 is occupied by a cysteine in a hydrophilic core. Its chain interacts with Thr40, Glu42, and, most importantly, the zinc finger in the normal protein, which are indicated in black. (**c**) A tyrosine with an aromatic nucleus in a hydrophobic core in the p.C41Y mutated protein, which has altered the chain conformation of the zinc finger, is shown in black.

Direct sequencing analysis of the *VDR* gene in the proband revealed a homozygous mutation, c.122G>A in exon2, resulting in a cysteine to tyrosine substitution at the 41th amino acid (C41Y) in the VDR protein. Both parents were found to be heterozygous for this mutation, and this mutation was not found in the 50 healthy controls (wild type) (Supplementary Figure 1). The C41Y mutation is located in the first zinc finger of the DBD (Figure 2).

## Discussion

Here we described a Chinese patient who presented with hypocalcemia, secondary hyperparathyroidism, hypophosphatemia, elevated ALP and 1,25(OH)_2_D levels, and rickets, consistent with a diagnosis of HVDRR. She also had early-onset alopecia and epilepsy. Her genetic analysis showed a homozygous mutation (C41Y) in the *VDR* gene, which had been first described by Shafeghati *et al.*^9^ in a Belgian family in 2008.

The VDR, a member of the steroid–thyroid–retinoid receptor gene superfamily of nuclear transcription factors, consists of: a conserved DBD (24–89 amino acids, domain C); a moderately conserved LBD (126–427 amino acids, domain E; which contains a dimerization interface and a ligand-dependent transcriptional activation domain); a connective hinge (domain D) between them; and a short A/B domain located at the N terminus.^3,11^ When vitamin D binds to the VDR, it changes its conformation to the active form and interacts with retinoic acid X receptor forming a heterodimeric complex (VDR/retinoic acid X receptor) that binds to vitamin D-responsive elements (VDREs) in the promoter regions of target genes.^8^ The conserved DBD zone has two zinc fingers, each of them containing four cysteine residues (24-cys, 27-cys, 41-cys, 44-cys, and 60-cys, 66-cys, 76-cys, 79-cys, respectively), which allows VDR to effectively recognize and bind the VDREs. When residues within this region are mutated, or when this domain is deleted, the receptor is normally produced but can no longer activate the VDREs.^12,13^ Consequently, the substitution of cysteine with a tryptophan located in the first zinc finger is most likely to interfere with VDR binding to VDREs in target genes and prevent the VDR complex form inducing target genes. In animal models of HVDRR, Bula *et al.*^14^ showed that mice that expressed a truncated VDR lacking the first zinc finger developed alopecia, demonstrating that an intact DBD is the critical requirement for hair follicle homeostasis, as well as for the prevention of alopecia. Subsequently, Malloy *et al.*^2^ demonstrated that a single point mutation in the conserved DBD zone can also cause alopecia. As previous studies have shown, a common feature of patients with DBD mutations is that they all have alopecia, either totalis or partialis. Similarly, our patient had normal hair at birth, but was found to have progressive loss of hair from the scalp and eyebrows to almost alopecia totalis within 12 months.

It is well recognized that vitamin D has critical roles in the intestine, kidney, parathyroid gland, and bone, regulating calcium and phosphate metabolism. Some studies indicated that in the absence of adequate amounts of the active hormones or a functional receptor (VDR), calcium, and phosphate absorption would be impaired, which leads to the disruption of calcium homeostasis resulting in hypocalcemia, hypophosphatemia, secondary hyperparathyroidism, and skeletal defects.^15,16^ In the present study, the patient with HVDRR showed the classical clinical manifestations of early-onset rickets, hypocalcemia, hypophosphatemia, elevated serum ALP and 1,25(OH)_2_D levels, and secondary hyperparathyroidism, implying the disruption of calcium and phosphate homeostasis. As previously reported,^2^ the major defects in HVDRR, such as the abnormal change of serum biomarkers and bone abnormalities, are mainly due to a defect in calcium absorption in the gastrointestinal tract. For these patients, phosphorus supplementation was not necessary and phosphate levels could be normalized along with the suppression of hyperparathyroidism with calcium supplementation alone. Thus, these reports indicate that the rachitic bone disease is mainly due to hypocalcemia, not PTH-driven urinary phosphate wasting and hypophosphatasia.

In addition to the classical clinical bone features, our patient suffered from epilepsy, which is a common disease of the central nervous system, but is rare in patients with HVDRR. Vitamin D is also a neuroactive steroid hormone with multiple functions in the nervous system, mediated by VDR, where they are widespread in the brain, implying that they may take part in the regulation of brain functions.^17,18^ In a mouse model with induced chemical seizures, Kalueff’s team directly linked the genetic ablation of VDR to increase seizure susceptibility and revealed that vitamin D has anti-convulsive activity in an epilepsy animal model.^17^ The findings in Kalueff’s study are consistent with the observations in patients with hypo-vitamin D and rickets, as well as our patient. Although the exact mechanism is still unclear, several potential physiological mechanisms have been hypothesized for the epilepsy caused by the functional incapacitation of VDR, as follows. (1) Epilepsy may be affected by *VDR* genetic ablation by disrupting VDR-mediated modulation of certain brain genes that may alter the baseline levels of endogenous proconvulsants and anticonvulsants, leading to the increase in epilepsy susceptibility.^18^ (2) Vitamin D has a role in stimulating the expression of Ca-binding proteins, which are also known to exert anti-epileptic effects. Thus, the genetic disruption of the *VDR* gene may affect the expression of VDR-mediated modulation of these proteins.^18^ (3) VDR has been recognized as a neuroprotective factor for a long time. The reduction of VDR-mediated neuroprotective function caused by *VDR* mutation may result in an increased susceptibility to seizures.^19^

Interestingly, the patient’s clinical manifestations improved markedly after 2 years of regular oral normal doses of vitamin D between 13 and 15 years of age. Previous data from patients with HVDRR revealed that calcium absorption is highly vitamin D dependent and only long-term oral supraphysiological doses of calcitriol and calcium, or intravenous injection of calcium, are able to maintain near-normal serum calcium levels in these patients from infancy to the end of puberty.^7, 8,20^ Our patient is obviously different from the cases in other studies. The restoration of calcium homeostasis with low doses of vitamin D and oral calcium could possibly be explained by the recent observations of VDR-independent mechanisms of intestinal calcium absorption. Some studies showed that estrogen could upregulate intestinal calcium absorption in VDR knockout mice and in humans by a VDR-independent mechanism.^21,22^ Thus, we surmised that in female adolescents with HVDRR, including our patient, a low dose rather than a high dose of oral calcium is sufficient to restore calcium homeostasis in the absence of 1,25(OH)_2_D signaling.^23,24^

To date, according to the published medical literature, 45 mutations have been identified in the *VDR* gene as the cause of HVDRR. Before our study, there has been only one compound heterozygous mutation in the *VDR* gene reported in Chinese people, that is, R80Q in exon 3 and N276Y in exon 7 by Malloy in 2014.

## Conclusions

In conclusion, we report a Chinese Han family with one individual affected with HVDRR and identified a homozygous missense mutation c.122G>A (p.C41Y) in the *VDR* gene. Compared with other patients with HVDRR who presented with hypocalcemia, hypophosphatemia, secondary hyperparathyroidism, and alopecia, our patient also had epilepsy and largely achieved calcium homeostasis with normal pharmacological doses of vitamin D. In contrast to previous reports of prepubescence patients with HVDRR, our patient obviously improved with supplementation of calcium and lower doses of active vitamin D, suggesting a wide spectrum of variety in this disease.

## Figures and Tables

**Table 1:** BMD of the patient with HVDRR (a 13-year-old girl)

Region	BMD/(g·cm^−2^)	*Z* score
L1–L4	0.815	0.21
Femoral neck	0.797	1.13
Total hip	0.952	−0.27

HVDRR, hereditary vitamin D-resistant ricket; BMD, bone mineral density.

The Z score of the patient was calculated by comparison with BMD measurements from age-matched Chinese children.

**Table 2:** Biochemical findings of the patient with HVDRR

Biochemical indicators	Before treatment	After treatment (2 years)	Reference range
Serum calcium/(mmol·L^−1^)	**1.85**	2.40	2.13–2.70
Serum phosphate/(mmol·L^−1^)	**0.80**	**1.28**	1.29–1.94
Serum alkaline phosphatase/(U·L^−1^)	**540**	141	58–400
Serum 25-hydroxyvitamin D/(ng·mL^−1^)	12.1	NA	5.0–50.0
Serum 1,25-dihydroxy vitamin D/(pg·mL^−1^)	**358.4**	**200.4**	19.6–54.3
Serum parathyroid hormone/(pg·mL^−1^)	**545.6**	**74.1**	12.0–65.0

HVDRR, hereditary vitamin D-resistant ricket; NA, not available.

Abnormal results are indicated in bold.
