# Supplementary material for: Clinical and genetic findings in a Chinese family with VDR-associated hereditary vitamin D-resistant rickets
Source: Bone Res. 2016 Jun 21;4:16018–. doi: 10.1038/boneres.2016.18 (PMC4923942; doi:10.1038/boneres.2016.18)
Supplement: Supplementary Table 1 [file boneres201618-s1.doc]

Table S1. The primer sequences used to amplify the exons 2-9 of the *VDR* gene

| Exons | Forward primers(5`~3`) | Reverse primers (5`~3`) | Anneal temperature (℃) | Size (bp) |
| --- | --- | --- | --- | --- |
| 2 | ACTGACTCTGGCTCTGACC | TCACTCTGGAAGGATGGAC | 58 | 502 |
| 3 | GCCTCATGTCTTCTGTTGGA | ACTCTTGAAGGCAGTGTCCG | 60 | 554 |
| 4-5 | TAAAGCCCCTCCTATCTTGGAC | AGTTTCCATTAGGGAGCCTTC | 60 | 772 |
| 6 | TTGATTTTACTGCCTTATG | ATAGTGAGCCAAGATAGTG | 54.5 | 738 |
| 7 | CCTACCTTTTGCAGACCACC | GTTCAGCTTCTTCAGTCCCAC | 59 | 761 |
| 8 | CGTGACCAAAGGTATGCCTA | ATCTAGTTCCTCAGAATCCCC | 59 | 582 |
| 9 | GGTCAGCAGTCATAGAGG | GCAGGAAAGGGGTTAG | 58 | 539 |
