# Supplementary figures and images for: Clinical and genetic findings in a Chinese family with VDR-associated hereditary vitamin D-resistant rickets
Source: Bone Res. 2016 Jun 21;4:16018–. doi: 10.1038/boneres.2016.18 (PMC4923942; doi:10.1038/boneres.2016.18)

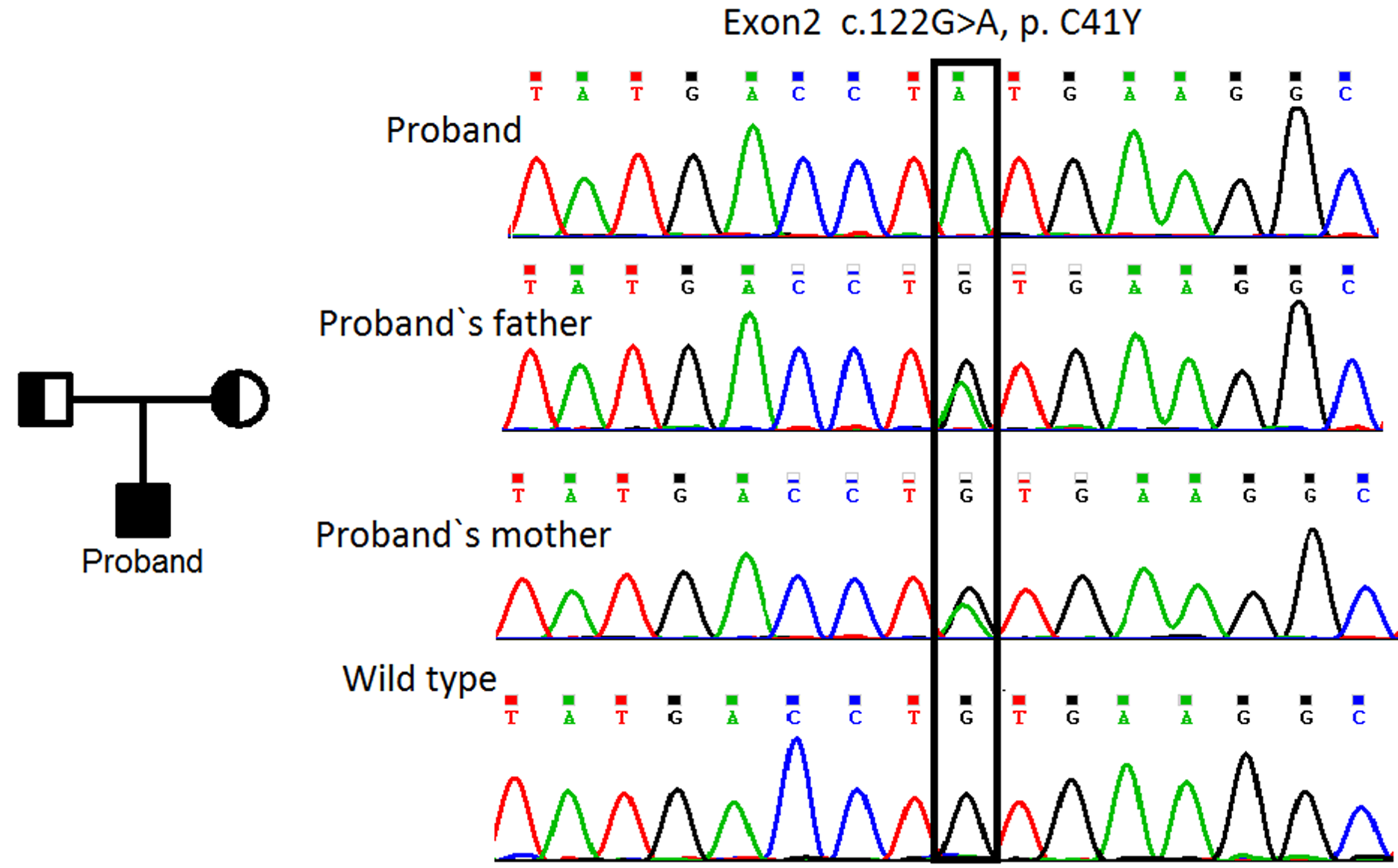

Supplement: Supplementary Figure 1 [file boneres201618-s2.tiff]
