# Supplementary material for: Clinical and genetic findings in a Chinese family with VDR-associated hereditary vitamin D-resistant rickets
Source: Bone Res. 2016 Jun 21;4:16018–. doi: 10.1038/boneres.2016.18 (PMC4923942; doi:10.1038/boneres.2016.18)
Supplement: Supplementary Figure Legends [file boneres201618-s3.doc]

Fig.S1. **Sequence analysis of the VDR gene in the proband and her parents**. Mutational analysis showed a homozygous mutation of the VDR gene in the proband, consisting of a single base G to A transition at cDNA nucleotide 122 in exon 2 (c.122G>A) that results in a cysteine to tyrosine substitution at the 41th amino acid (p.C41Y) in theVDR protein. Her parents were both asymptomatic heterozygous carriers for this mutation. This mutation was not found in 50 healthy controls (wild type) (indicated in the black box). The family pedigrees are shown in the left, the patient with HVDRR is indicated with dark symbols, and carriers are identified with half-dark symbols.
